# Novel Analysis Software for Detecting and Classifying Ca^2+^ Transient Abnormalities in Stem Cell-Derived Cardiomyocytes

**DOI:** 10.1371/journal.pone.0135806

**Published:** 2015-08-26

**Authors:** Kirsi Penttinen, Harri Siirtola, Jorge Àvalos-Salguero, Tiina Vainio, Martti Juhola, Katriina Aalto-Setälä

**Affiliations:** 1 BioMediTech, University of Tampere, Tampere, Finland; 2 School of Medicine, University of Tampere, Tampere, Finland; 3 Tampere Unit for Computer-Human Interaction, University of Tampere, Tampere, Finland; 4 Research Center for Information and Systems, University of Tampere, Tampere, Finland; 5 Heart Center, Tampere University Hospital, Tampere, Finland; University of Newcastle, AUSTRALIA

## Abstract

Comprehensive functioning of Ca^2+^ cycling is crucial for excitation–contraction coupling of cardiomyocytes (CMs). Abnormal Ca^2+^ cycling is linked to arrhythmogenesis, which is associated with cardiac disorders and heart failure. Accordingly, we have generated spontaneously beating CMs from induced pluripotent stem cells (iPSC) derived from patients with catecholaminergic polymorphic ventricular tachycardia (CPVT), which is an inherited and severe cardiac disease. Ca^2+^ cycling studies have revealed substantial abnormalities in these CMs. Ca^2+^ transient analysis performed manually lacks accepted analysis criteria, and has both low throughput and high variability. To overcome these issues, we have developed a software tool, *AnomalyExplorer* based on interactive visualization, to assist in the classification of Ca^2+^ transient patterns detected in CMs. Here, we demonstrate the usability and capability of the software, and we also compare the analysis efficiency to manual analysis. We show that *AnomalyExplorer* is suitable for detecting normal and abnormal Ca^2+^ transients; furthermore, this method provides more defined and consistent information regarding the Ca^2+^ abnormality patterns and cell line specific differences when compared to manual analysis. This tool will facilitate and speed up the analysis of CM Ca^2+^ transients, making it both more accurate and user-independent. *AnomalyExplorer* can be exploited in Ca^2+^ cycling analysis to study basic disease pathology and the effects of different drugs.

## Introduction

Calcium (Ca^2+^) cycling plays an essential role in the excitation-contraction coupling of cardiomyocytes (CMs) and is therefore vital for cardiac functionality. However, cardiac diseases and different drugs can cause changes and variability in Ca^2+^ cycling that can affect the function and phenotype of CMs. Characterizing these disturbances and abnormalities is vital to improving the studies of the disease pathology and disease prevention and treatment. Intracellular Ca^2+^ cycling of CMs is analyzed *in vitro* with Ca^2+^ sensitive dyes that change their fluorescence properties when binding to Ca^2+^ ions.

Induced pluripotent stem cell (iPSC) technology, in which pluripotent stem cells are generated by reprogramming differentiated cells back into the pluripotent state, provides a method for studying the pathophysiology of various disorders in human cells.[1] iPSCs can be differentiated into the desired cell type and retain the original genotype. New insights into the Ca^2+^ cycling of different cardiac diseases have been achieved since the invention of iPSC technology. The functionality of CMs and their drug responses could be studied more thoroughly with investigation of Ca^2+^ transients. Disease modeling with iPSCs has been successfully exploited to study cardiac diseases such as catecholaminergic polymorphic ventricular tachycardia (CPVT) [2–9], dilated cardiomyopathy [10], hypertrophic cardiomyopathy [11,12] and Timothy syndrome [13]. In these studies, Ca^2+^ cycling analyses have revealed substantial defects and abnormalities in CMs that reflect the cardiac phenotype observed in patients. However, analyzing these Ca^2+^ transient abnormalities, which consist of cycling patterns that vary in shape and frequency, is difficult, and their quantification is very challenging. To the best of our knowledge, Ca^2+^ cycling abnormality patterns have thus far only been subjectively analyzed based on visual information, which is labor-intensive, slow and often user-dependent. Manual data analysis performed with visual recognition is also a bottleneck for high-throughput screening and can occasionally be unreliable because of the poor quality of the Ca^2+^ transients. Having general criteria for Ca^2+^ cycling abnormality analysis could simplify the comparison between different studies and facilitate the analysis of Ca^2+^ cycling parameters. In addition, an automated Ca^2+^ cycling classification software would be beneficial for the future analysis of Ca^2+^ cycling of CMs on a large scale to screen for adverse cardiac effects of new potential compounds.

The aim of the study was to develop and test a Ca^2+^ cycling abnormality analysis software based on interactive visualization with a direct manipulation user interface (UI). The software, *AnomalyExplorer*, can user-independently assist in the classification of the Ca^2+^ transient patterns detected in CMs by using criteria that are similar to those used in manual analyses that are performed with visual recognition. We demonstrate the usability and capability of the software using iPSC-derived CMs that were generated from patients with cardiac ryanodine receptor (RyR2) gene mutations causing CPVT. Opening of RyR2 Ca^2+^ channels underlies intracellular Ca^2+^ release from the sarcoplasmic reticulum in CMs. CPVT is an exercise-induced arrhythmogenic cardiac disease with intracellular Ca^2+^ cycling defects [14]; therefore, CPVT-specific CMs [5,9] are suitable for evaluating this program. The usability of the *AnomalyExplorer* is demonstrated with the data generated by two different Ca^2+^ imaging recording software programs with different sampling frequencies. The analysis of data using *AnomalyExplorer* is compared with the manual analysis results to establish the reliability of the analysis software. This software tool will improve the accuracy, reliability and throughput rate for Ca^2+^ cycling data analysis.

## Materials and Methods

### Generation of patient-specific iPSCs and cardiac differentiation

The study was approved by the ethical committee of Pirkanmaa Hospital District (R08070). Patient-specific iPSC lines were established as described earlier and written informed consent was obtained from all the participants.[1] The studied RyR2 mutated CPVT cell lines were UTA.05605.CPVT, generated from a patient with exon 3 deletion (c.168–301_c.273+722del1128 mutation); UTA.05208.CPVT, generated from a patient with a p.P2328S (c.6982C>T) mutation; UTA.07001.CPVT, from a patient with a p.T2538R (c.7613C>G) mutation; UTA.03701.CPVT, from a patient with a p.L4115F (c.12343C>T) mutation; UTA.05503.CPVT, from a patient with a p.Q4201R (c.12602A>G) mutation; and UTA.05404.CPVT, from a patient with a p.V4653F (c.13957G>T) mutation. UTA.04602.WT cell line was generated from a healthy control. Mutation nomenclature was based on RyR2 reference sequence NM_001035.2. The results of the characterization of iPSC lines have been previously described.[5,9] For illustration of the RyR2 protein and locations of the mutations, see [9].

Differentiation into CMs was performed by co-culturing iPSCs with murine visceral endoderm-like (END-2) cells (Humbrecht Institute, Utrecht, The Netherlands) as previously described.[15] For Ca^2+^ cycling measurements, CMs were dissociated by mechanically excising the beating areas of the cell colonies and treating them with collagenase A (Roche Diagnostics) [15].

### Ca^2+^ imaging recordings and data analysis

Ca^2+^ imaging was conducted in spontaneously beating Fura-2 AM (Life Technologies, Molecular Probes) loaded, dissociated CMs that were perfused with extracellular solution as previously described.[5] Ca^2+^ measurements were performed on an inverted IX70 microscope (Olympus Corporation, Hamburg, Germany) and CMs were visualized with a UApo/340 x20 air objective (Olympus). Images were recorded with an ANDOR iXon 885 CCD camera (Andor Technology, Belfast, Northern Ireland) that was synchronized with a Polychrome V light source by a real-time DSP control unit and TillVision (TILL Photonics, Munich, Germany) or Live Acquisition software (FEI, OR, USA). TillVision and LiveAcquisition are further referred as Recording Software 1 and 2, respectively. Fura-2 AM in CMs was excited at 340 nm and 380 nm light and the emission was recorded at 505 nm. For Ca^2+^ analysis, regions of interest were selected for spontaneously beating cells and background noise was subtracted before further data processing. The Ca^2+^ transients were acquired as the ratio of the emissions at 340/380 nm wavelengths and presented as Fura-2 ratio units of F340/F380. Throughout the remainder of this manuscript, the Ca^2+^ recording of a single cell is called the Ca^2+^ signal.

The data were generated with two separate softwares (Recording Software 1 and 2) with different sampling frequencies that affected the noise level of the recordings. Therefore, the Ca^2+^ signals were divided into two groups and analyzed separately depending on the recording software. The Ca^2+^ signals were manually analyzed with visual recognition by identifying the signals as normal or abnormal and categorizing the abnormalities into six subgroups according to their abnormality patterns. In manual analysis, a criterion for the double peak group was a Ca^2+^ peak with paired peaks that did not reach the baseline. Oscillation was detected if Ca^2+^ fluctuated for three or more peaks without reaching the baseline. For the low peaks group, the presence of a small amplitude Ca^2+^ peak of at least 10% of the preceding Ca^2+^ peak amplitude was required. In the middle Ca^2+^ peaks, 30 to 80% amplitude of the preceding Ca^2+^ peak amplitude was required. Plateau abnormality was detected if the rise or decay time of the Ca^2+^ peak was prolonged, and an irregular beating rhythm was required for the irregular phase group. Here, the Ca^2+^ peak represents a Ca^2+^ transient consisting of Ca^2+^ rise and decay. For comparison studies, the Ca^2+^ data was analyzed with *AnomalyExplorer*, in which suitable user-defined percentage limits for each abnormality subgroup were selected for both recording software data groups.

### Software tool description

*AnomalyExplorer* is an interactive software tool that can assist in the visual classification of Ca^2+^ signals. It is based on the information visualization approach with the following two major components: representation of data and interaction with the data. The representation component of *AnomalyExplorer* is based on the detail+overview design pattern, and an overview and detailed view of an information space are displayed simultaneously. Generally, the overview provides a high-level view of the information space, and the detailed view shows the current focus of interest in full detail.

The detailed view in *AnomalyExplorer* is a conventional 2D plot of the signal value over time. The plot is similar to what a human observer would use to detect anomalies in the shape of a signal. The idea in *AnomalyExplorer* is to express the human observer’s criteria for abnormality as rules that can be programmed into *AnomalyExplorer*. The tool then highlights the detected anomalies in a plot, which allows the user to adjust the sensitivity of anomaly detection by user-defined parameters for signal noise and six different abnormality subgroups.

The most important qualitative aspect in *AnomalyExplorer* is to define whether the signal is normal or not, and to further analyze the quality and distribution of detected anomalies. Thus, the overview in *AnomalyExplorer* was designed to be a stacked bar chart of detected anomalies where a bar represents the number of anomalies in a signal, and stacking shows how the anomalies are distributed. Only the abnormal signals are present in the bar chart because the signals deemed normal are not of interest. Information in such a bar chart can be acquired rapidly, and the conventional representation allows for further insight-generating interactions.

#### User interface (UI)

**Fig 1:**
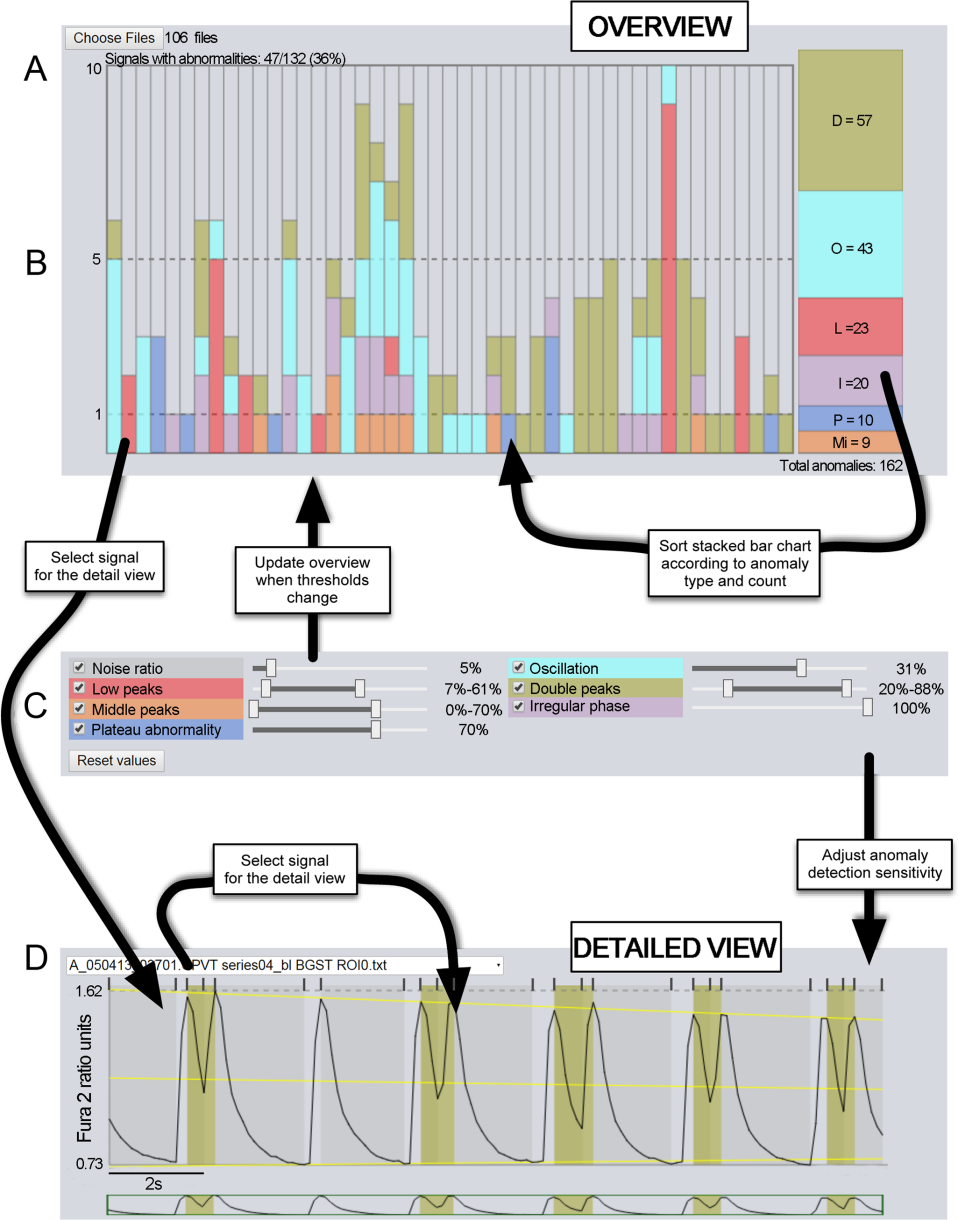
*AnomalyExplorer* program. A) Overview of the software. B) Each bar presents a single Ca^2+^ signal of a single cell and shows the distribution of abnormality patterns in the recording. The total number of anomalies in all of the files can be observed on the right. C) Dynamic controllers for the anomaly detection. D) The detailed view of one Ca^2+^ signal plot showing color-coded anomalies detected with the help of three yellow regression lines (top, bottom and middle) and the sections subsequently rising and decaying separated with small grey vertical lines above the signal showing how the Ca^2+^ transients ascend (light bars) and descend (dark bars).

Fig 1 shows the UI of *AnomalyExplorer* and illustrates the workflow. The current UI design has the following elements: a file chooser to open the signals that will undergo analysis, the stacked bar chart of observed anomalies (overview), dynamic controllers for the anomaly detection sensitivity, and the currently open set of detailed signal views. After these main components there is a section showing how the currently open signals are classified as normal and abnormal (as two text lists, not shown in the Fig 1), and a text view summarizes the anomalies found in the current set.

The designed workflow with *AnomalyExplorer* starts by choosing the signal files that will undergo analysis. The open file dialog allows for the selection of a single file, multiple files, or all of the files in the current directory. *AnomalyExplorer* then updates the overview to show the anomalies with the current default settings. Subsequently, the user may open one or more signals in the detailed view for closer inspection by clicking on a file name of the signal above the detailed view (Fig 1D) or if the signal includes anomalies, also by clicking a bar in the overview (Fig 1B). User can also adjust the dynamic controllers for the anomaly detection, observe the changes in both the overview and detailed view, and sort the overview according to any of the abnormality subgroup. The manipulation of dynamic controllers is immediately reflected in both the overview and detailed view and allow for a rapid what-if exploration of anomaly thresholds. When the satisfactory user-defined parameters for anomaly detection of a certain data set are found, the *AnomalyExplorer* tool can be used as a rapid user-independent classifier. The preset values of dynamic controllers serve only as a starting point to seek the best parameter set for the current data set. Once the parameter values that correspond the analyst’s view of the anomalies are found, the presets can easily be made default to facilitate easy classification of multiple files without changing the parameters.

#### Anomaly detection

*AnomalyExplorer* is designed to detect the anomalies in the same fashion as a human observer. The automated analysis software is based on the concepts of sections and regression lines, which act as points of reference for the rules defining the anomaly subgroups. A section is a part of a signal which ascends or descends monotonously; it thus marks and separates the Ca^2+^ rise and decay, which together comprise a Ca^2+^ transient. Sections allow for a small amount of noise in the signal. The noise threshold determines how much the signal may exhibit direction changes without considering it to be a change in monotonous movement. The regression lines are needed as a reference for the anomaly rules because the amplitude of a Ca^2+^ signal may fade over time from the Ca^2+^ indicator photobleaching. The software uses the following three regression lines (Fig 1): one that approximates the signal’s local maximum (in a transient, top), another for signal’s local minimum (bottom), and a third one that is averaged from these two, which approximates the local middle of the signal (middle). The computation of the simple linear regression lines is based on the local maximum and minimum data values in each section, and it is meant to approximate how a human observer would place them. In addition to the regression height, the section heights are also used in the computations. The section height is simply the data range in the section (i.e., the local maximum data value minus the local minimum data value), and the regression height is the distance between the top and bottom regression lines in the current section. With these reference values the anomaly subgroups can be defined. In addition, the anomaly definitions refer to predefined and user-defined parameters. The predefined ones are implementation-dependent constants and the user-defined are values that can be manipulated via the UI to correspond to Ca^2+^ measurement settings such as the frame-rate of the recordings.

**Fig 2:**
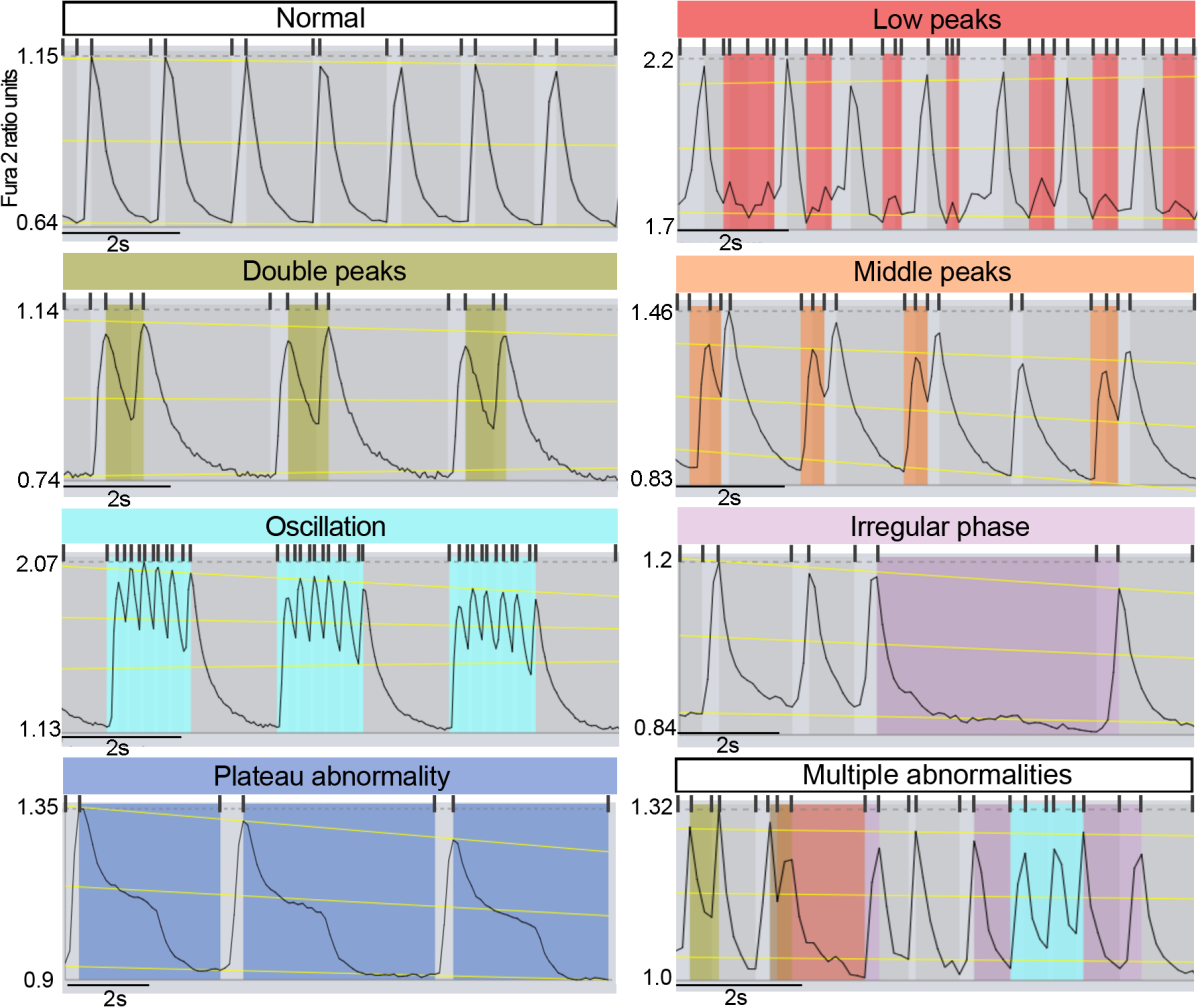
Detected Ca^2+^ signal abnormality patterns from *AnomalyExplorer* analysis. Examples of the color-coded abnormality subgroups that *AnomalyExplorer* detects as well as normal Ca^2+^ signals as follows: low peaks, double peaks, middle peaks, oscillation, irregular phase, plateau abnormality, and a signal with multiple different abnormalities. Abnormalities are detected with the help of three yellow regression lines and subsequently rising and decaying sections separated with small grey vertical lines above the signal showing how the Ca^2+^ transient ascends and descends monotonously. The same abnormality patterns were detected in a manual analysis made with visual recognition.

The current implementation of *AnomalyExplorer* detects the following six abnormality subgroups: low, middle, oscillating and double peak anomalies as well as irregular phase and plateau abnormality (Fig 2).[16] Each of these anomaly subgroups are described in more detail below. User-defined parameters can be manipulated by using the dynamic controllers in the UI.

An ascending signal section is flagged to have a *low peak* (Fig 2) if the following conditions are met: 1) the transient minimum value is within 15% of the local regression height from the local bottom regression line, 2) the transient maximum value is between user-defined percentage limits of the local regression height, and 3) the previous and the following transient do not contain double peak anomalies. Once the ascending section is classified as a low peak, the following descending section is also flagged as a low peak if its minimum value is within ±10% of the current section’s minimum, i.e., the peak is fairly symmetrical.

The *middle peak anomaly* (Fig 2) is flagged if the following conditions are met: 1) the section height is within the user-defined percentage limits of the local regression height, 2) the transient maximum value is within 60% of the local middle regression line, 3) the transient minimum value is within 40% of the local middle regression line, 4) the section height is less than 75% of the height of the surrounding sections, and 5) there is no low peak anomaly in the previous or this section.

When determining the *oscillation* (Fig 2) of the Ca^2+^ signal the software compares the section and transient heights and lengths, and the consecutive transients that are dwarfed below the user-defined percentage limit are flagged as oscillating. The oscillating transients are not analyzed for any further anomalies. Oscillations can also be generated by a double peak anomaly, which is discussed below.

A *double peak anomaly* (Fig 2) is flagged if the transient does not descend sufficiently low before ascending to the next peak, such that only the descending sections are considered. The double peak anomaly is flagged if the following conditions are met: 1) the section maximum value is within 40% of the section height of the local top regression line, 2) the height of the section is within user-defined percentage limit of the local regression height, 3) the upper percentage limit determines how deep the double peak can be (where 100% is the local bottom regression line and 50% is the local middle regression line), and 4) the height of the section is within ±25% of the height of the following section. There can only be two consecutive peaks in a double peak anomaly and sequences of more than two peaks are flagged as oscillations.

*Irregular phase* (Fig 2) is flagged if the distance of peaks differs by a user-defined percentage (for example, 90%) from the median of the peak distances. Only those peaks that do not exhibit any anomalies are considered, except the peaks that exhibit double peaks anomaly which are treated as a single peak that has the mean of the positions of double peaks as its reference value. Treating a double peak as a single peak prevents the overlap of irregularity and double peak anomaly.

*Plateau abnormality* (Fig 2) is flagged if the transient changes its rate of ascent or descent more than a user-defined percentage (for example 75%) within a section. Its recognition is also affected by the setting of the noise ratio, i.e., how much of the signal noise is tolerated.

#### Availability of Software

The *AnomalyExplorer* conforms to the Open Source Definition and has been designed to facilitate the abnormality analysis of Ca^2+^ recordings obtained from CMs. Website for downloading the program: https://github.com/siirtola/AnomalyExplorer. More information about the software can be found from S1 Supporting Information.

## Results

Ca^2+^ transient abnormality patterns of CMs derived from iPSC lines with RyR2 mutations causing CPVT varied remarkably as a function of time. We investigated the reliability and efficiency of the *AnomalyExplorer* in detecting the normal and abnormal Ca^2+^ transients and categorized the abnormal signals into pattern specific subgroups, and compared these results to the manual analysis.

The data were generated with two different Ca^2+^ transient recording software programs. As a result, the signals were recorded with various sampling frequencies, which ranged from 9 to 10 Hz on Recording Software 1 and from 23 to 26 Hz on Recording Software 2. This affected the noise level of the recordings, and it was thus necessary to find the optimal *AnomalyExplorer* user-defined anomaly detection parameters for both recording software groups. In total, 132 Ca^2+^ signals were recorded with Recording Software 1 and 212 Ca^2+^ signals with Recording Software 2, and the lengths of recordings varied from approximately 11 to 23 s.

Each Ca^2+^ signal was manually analyzed with visual recognition and compared with the results obtained from *AnomalyExplorer*. On both manual and *AnomalyExplorer* analysis, the Ca^2+^ signals were categorized as abnormal if at least one abnormality pattern was visualized in the signal and as normal if no abnormalities were found in the recordings. Abnormal signals were further categorized into different subgroups based on the following types of Ca^2+^ abnormalities observed visually: double peaks, oscillating peaks, low amplitude peaks, plateau abnormalities with prolonged Ca^2+^ rise or decay time or irregular phase in the beating rhythm (Fig 2). Then, the total numbers of subgroup specific abnormalities were quantified from the Ca^2+^ signals. One signal could belong to more than one abnormality subgroup and could include several abnormalities.

At first, in *AnomalyExplorer* analysis, optimal analysis parameters were found for both recording software programs. For signals generated with Recording Software 1 the user-defined percentage limits for analysis were the following: noise 5%, low peaks 7–61%, middle peaks 0–70%, plateau abnormality 70%, oscillation 31%, double peaks 20–88%, and irregular phase 100%. For signals generated with Recording Software 2, the user-defined percentage limits were the following: noise 8%, low peaks 10–61%, middle peaks 0–70%, plateau abnormality 70%, oscillation 31%, double peaks 20–88%, and irregular phase 100%. When comparing the detection of normal and abnormal Ca^2+^ signals between the manual analysis and *AnomalyExplorer* (S1 Fig), there was only 2% inconsistency between the manual and *AnomalyExplorer* analysis for Recording Software 1 signals. This inconsistency was specified as false abnormal, which indicated that they were manually categorized as normal but that the *AnomalyExplorer* categorized them as abnormal. For Recording Software 2 signals, there was 9% inconsistency with manual analysis; 4% were false abnormal and 5% were false normal, which indicated that they were manually categorized as abnormal but that the software categorized them as normal.

**Fig 3:**
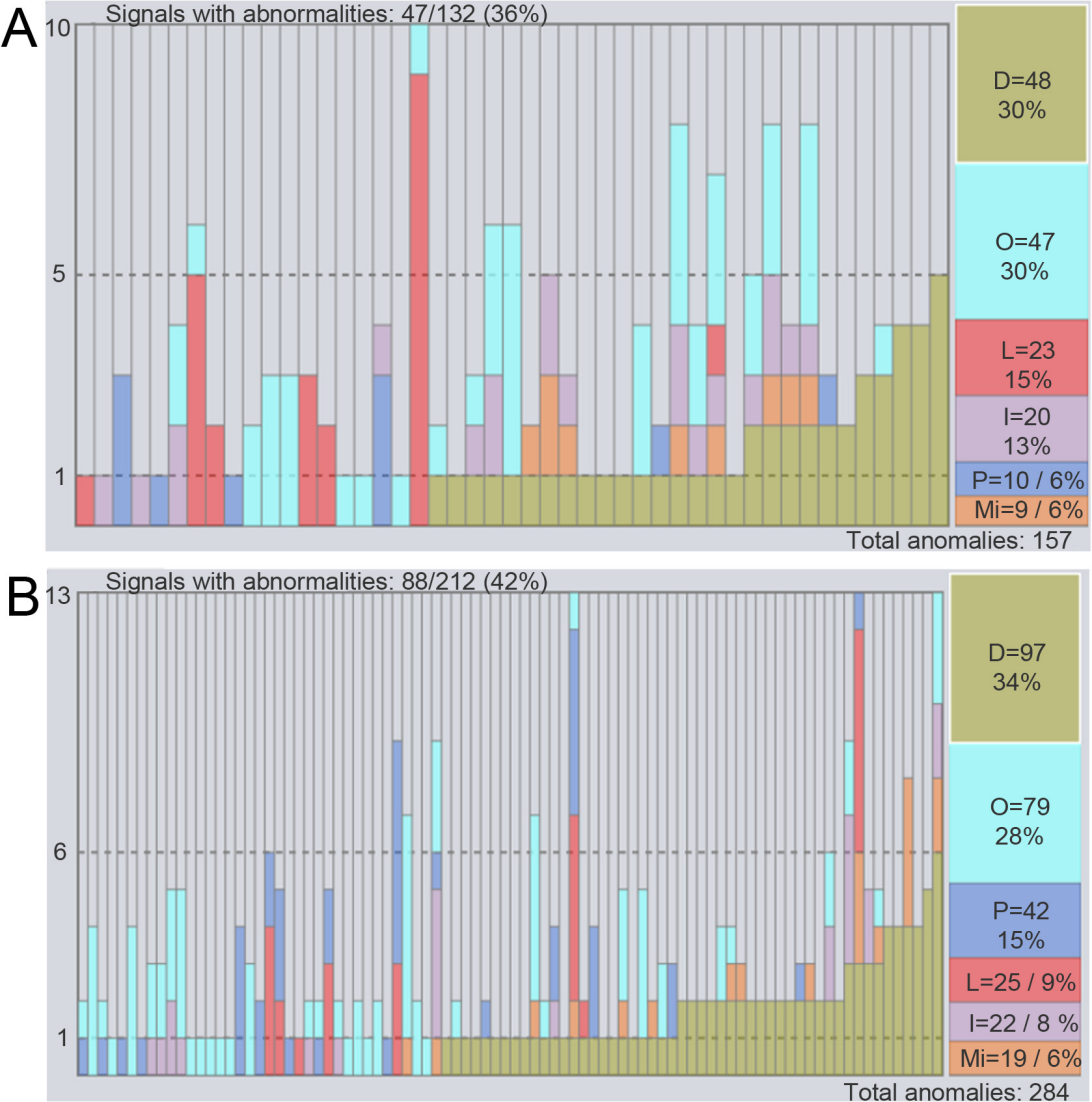
Detailed results in the *AnomalyExplorer* overview. View of the *AnomalyExplorer* analyzed Ca^2+^ signals summarizing the results recorded with A) Recording Software 1 and B) Recording Software 2. It shows the detailed number of subgroup specific abnormalities in each Ca^2+^ signal in one bar and the total number and percentages of all abnormality subgroups. Each bar represents a single cell (signal) and only signals presenting with abnormalities are shown. Altogether, 132 Ca^2+^ signals were recorded with Recording Software 1, 47 which (36%) presented abnormalities. The corresponding number from 212 Ca^2+^ signals recorded with Recording Software 2 was 88 (42%).

The majority of the spontaneously beating control WT and CPVT CMs were categorized as normal in both the manual analysis (66% and 58% of Recording Software 1 and 2 signals, respectively) and the *AnomalyExplorer* analysis (64% and 57% of Recording Software 1 and 2 signals, respectively), without any observed abnormalities. Overall, 17% of the control WT CMs (n = 23) were categorized as abnormal with *AnomalyExplorer* analysis and 13% with manual analysis. Table 1 summarizes the overall percentage of detected abnormal signals with both recording software programs as well as the consistency of the manual and *AnomalyExplorer* analysis of different Ca^2+^ transient abnormality subgroups. To visualize the same results, see S2 Fig. Many signals that were categorized as abnormal presented with various Ca^2+^ transient abnormalities, which can be observed from the detailed analysis results in the *AnomalyExplorer* overview in Fig 3.

**Table 1:** Comparison of detected Ca^2+^ transient abnormalities and different abnormality subgroups analyzed manually and with *AnomalyExplorer* software. Each analyzed signal represents a recording from a single cell. The percentages show the amount of each subgroup specific abnormality when compared to the amount of all the abnormalities in all the signals. One signal could belong to more than one abnormality subgroup and could include several abnormalities. Last row of the table shows the overall percentage of the abnormal signals from all the analyzed signals. The signal was analyzed as abnormal if there were one or more Ca^2+^ transient abnormalities in the whole signal.

	Recording Software 1		Recording Software 2	
	Manual analysis, abnormalities (n = 171)	AnomalyExplorer analysis, abnormalities (n = 157)	Manual analysis, abnormalities (n = 301)	AnomalyExplorer, analysis abnormalities (n = 284)
Double peaks	29%	30%	33%	34%
Low peaks	20%	15%	15%	6%
Middle peaks	5%	6%	9%	9%
Oscillations	26%	30%	26%	28%
Plateau abn.	6%	6%	7%	15%
Irregular phase	14%	13%	13%	8%
Signals categorized as abnormal	34% (n = 132)	36% (n = 132)	43% (n = 212)	42% (n = 212)

**Fig 4:**
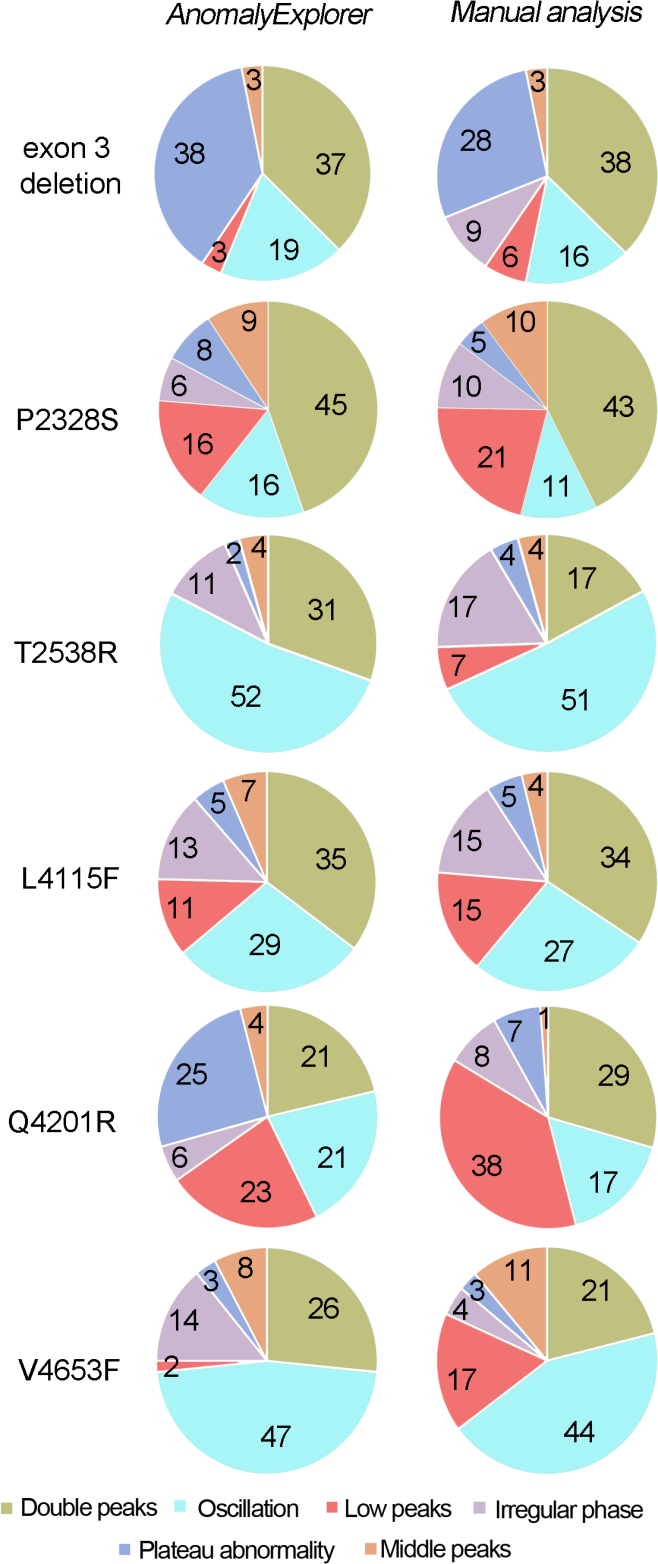
Comparison of *AnomalyExplorer* and manual Ca^2+^ signal analysis of the different RyR2 mutated CPVT cell lines. Pie charts indicate the percentage of the Ca^2+^ cycling abnormalities and show mutation specific differences in the abnormalities with both analysis methods. One Ca^2+^ signal can belong to several subgroups and consist of many abnormalities. The numbers of the signals analyzed from different RyR2 mutation specific CMs are the following: exon 3 del n = 46, P2328S n = 32, T2538R n = 51, L4115F n = 106, Q4291R n = 57 and V4653F n = 29.

When comparing the manual and *AnomalyExplorer* analysis of different RyR2 mutated CPVT cell lines, analyses revealed that there were mutation-specific differences in the Ca^2+^ transient abnormalities with both analysis methods (Fig 4). Double peaks were common abnormality types in all RyR2 mutated CPVT cell lines with some small differences between mutations. In *AnomalyExplorer* analysis double peaks were seen in 31 to 37% of the signals with mutations located with N-terminal or central cytosolic region of the *RyR2* protein (exon 3 deletion, P2328S, T2538R, L4115F) and in 21 to 26% of the signals with mutations in or near the transmembrane domain (Q4201R and V4653F). Oscillation was also common abnormality type in all of the mutated cell lines in *AnomalyExplorer* analysis, particularly in V4653F and T2538R mutated CMs (47% and 52%, respectively). Low peaks were rather common in P2328S and Q4201R mutated CMs (16% and 23%, respectively), and plateau abnormalities in Q4201R and exon 3 deletion mutated CMs (25% to 38%, respectively) in *AnomalyExplorer* analysis. Middle peaks and irregular phases were least common abnormality types in all mutated CPVT CMs. When comparing the consistency of the manual and *AnomalyExplorer*, it was shown that in P2328S and L4115F mutated CMs the analyses were comparable. Over 5% difference between analyses was seen in low peak abnormalities (with exon 3 deletion, T2538R and V4653F mutated CMs) with difference of 7–15%, irregular phase abnormalities (with exon 3 deletion, T2538R and V4653F mutated CMs) with difference of 6–10%, plateau abnormalities (with exon 3 deletion and Q4201R CMs) with difference of 10–18% and double peak abnormalities (with T2538R and Q4201R CMs) with difference of 8–14%.

## Discussion

Ca^2+^ cycling is fundamental for the electrical signaling and mechanical contraction of the heart. It is controlled via ion currents, channels, and exchangers. Comprehensive functioning of Ca^2+^ cycling is essential for the excitation–contraction coupling of CMs.[17] Ca^2+^ cycling abnormalities may cause arrhythmic features such as delayed after depolarizations (DADs), where depolarizing oscillations in the membrane potential follow an action potential after completion of repolarization [18], or early afterdepolarizations (EADs) which occur during repolarization.[19–21] Dysfunction in Ca^2+^ cycling may also produce Ca^2+^ alternans, a beat-to-beat alternation in the amplitude of the intracellular Ca^2+^ transient.[22] Previous studies have shown that disease-specific iPSC-derived CMs with Ca^2+^ cycling abnormalities manifest arrhythmic features such as DADs or EADs in their action potential [2–6,11,13]. Additionally, simultaneous AP and Ca^2+^ recordings of long QT-specific CMs have revealed that Ca^2+^ cycling is involved in prolonging the AP duration and in the formation of EADs as demonstrated by comparable changes in the APs and Ca^2+^ transients.[23] Therefore, disease phenotype-specific changes in electrophysiology can be mirrored by changes in Ca^2+^ cycling, thereby highlighting the importance of the analysis of these transients and abnormalities.

After the invention of iPSC technology, disease modeling with patient-specific CMs has been constantly increasing and has revealed Ca^2+^ transient abnormalities in many CM disease phenotypes. Consistent rules for the analysis of Ca^2+^ transient abnormalities are currently lacking; thus, the results obtained from different studies cannot be compared. Thus far, the detection of Ca^2+^ cycling abnormalities has been based on how the researchers categorize data based on the individual’s reasoning, experience and observations. Previously in our study and in other studies, earlier classification methods have involved categorizing a Ca^2+^ signal as abnormal if at least one abnormality is detected without indicating the absolute amount or duration of Ca^2+^ abnormalities in each signal.[3–6,11,12] In some cases, abnormalities have been divided into subgroups that do not necessarily consider that one signal can express different abnormal patterns and can thus belong to several subgroups.[4–6] A common problem has also been that the naming and/or illustration of the abnormalities differ between studies and they have been categorized, for example as after-contractions or triggered contractions [3], as significant Ca^2+^ transient irregularities, such as multiple events [11], or as significant irregularities in Ca^2+^ transients, including at least an irregular rhythm and multiple peaks.[12] Additionally, no specific analysis criteria have been reported because the nature of the analysis has been visual inspection. Therefore, it is unclear whether all of the patterns have been calculated as abnormalities and how these abnormalities have been considered in the numerical analysis of the data. Additionally, with the traditional method of presenting signal abnormalities, it is not possible to identify disease-specific Ca^2+^ abnormality patterns when comparing the already published reports.

The human visual system is an ingenious pattern recognizer, and our cognitive activity is largely based on this capability.[24] Detecting patterns is easy for a human, and the brain does it effortlessly on the subconscious level. For example, the activity of analyzing and classifying signals is trivial to those who know what to look for. However, regardless of the ability to categorize patterns manually, it is highly subjective. Two people might make different classifications, and the same person might classify signals differently at different times. Therefore, there is a huge demand for specific criteria for different subgroups of Ca^2+^ abnormalities with specific analysis software that could automatically classify and calculate the abnormal signals according to preset specifications. For these reasons, *AnomalyExplorer*, an interactive software tool that can assist in the visual classification of Ca^2+^ signals was developed to help researchers working with Ca^2+^ data of CMs. *AnomalyExplorer* is based on interactive visualization with a direct manipulation UI, in which the analysis and classification approach of the researcher has been transferred into a software tool to improve the accuracy, reliability and throughput rate for Ca^2+^ cycling analysis. The user can set the user-defined anomaly detection parameters for each subgroup and the background noise level of the recordings. This direct manipulation of the UI allows for the use of *AnomalyExplorer* with different measurement settings wherein the recording software, sampling frequency or environment of the measurement setup can vary and optimal settings can be found for each occasion. Once the optimal user-defined anomaly detection parameters have been identified for recordings made with specific measurement settings, there is no need to manipulate the UI.

The purpose of information visualization is to use perception to amplify cognition. The idea of *AnomalyExplorer* was to generate task-specific visual representations of data with which the user can interact, thereby amplifying users’ cognition. The means to amplify users’ cognition are numerous, and here the most relevant ones were enhancing the pattern recognition, facilitating the comparisons, and supporting the short-term memory by using external representations. [25] The interaction component of the software allowed the user to have a dialog with the data, and this exploration uncovered insights.[26] The representation component of *AnomalyExplorer* was based on the detail+overview design pattern, and an overview and detailed view of an information space are displayed simultaneously.[27] Overview has been defined to imply a qualitative awareness of one aspect of some data, preferably acquired rapidly and pre-attentively without cognitive effort. [28] The most important qualitative aspect in *AnomalyExplorer* was to define whether the signal is normal or not, and to further analyze the quality and distribution of detected anomalies. Therefore overview in *AnomalyExplorer* was designed to be a stacked bar chart of detected anomalies where a bar represents the number of anomalies in a signal, and stacking shows how the anomalies are distributed. The first question in the design of an interactive visualization tool is if often whether we need to support a workflow from the overview to detailed view or vice versa. Shneiderman [29] advocated for the former approach in designing visualization tools by stating “overview first, zoom and filter, then details-on-demand”. In *AnomalyExplorer*, the free movement between the overview and detailed views must be supported to reduce the cognitive load. In addition, the views need to be tightly coupled. In the detailed view, the appropriate parameters for anomaly detection are sought, and it must be continuously monitored how this affects the overall outcome, until suitable parameters for data analysis are found. In the overview, the inspection may reveal signals that seem to be outliers or that are otherwise suspicious, and the details on the demand need to be seen.

It can be roughly estimated that manual analysis of one Ca^2+^ signal takes from 10 to 120 seconds, and the overall manual analysis time in this study was approximately 90 minutes and 140 minutes with Recording Software 1 and 2 signals, respectively. With *AnomalyExplorer*, the analysis of the Ca^2+^ signals is much faster, and was approximately only 10 seconds in this study with both Recording Software 1 and 2 signals, because the analysis results are immediately shown after loading the signals into the software regardless of the number of signals. Therefore, with *AnomalyExplorer*, the saved time compared with manual analysis increases with the number of analyzed signals. Other benefits of *AnomalyExplorer* include the fact that the classifications are repeatable and user-independent and that the accuracy and repeatability of the analysis can be enhanced. The definition of signal abnormalities in terms of visual properties, is more flexible than numerical or analytical specification. However, it needs to be highlighted that this type of abnormality analysis does not exclude the need for the numerical analysis of different Ca^2+^ cycling parameters, such as the amplitude, frequency and peak duration. AnomalyExplorer is anyhow a good tool for screening through the Ca^2+^ recordings for more extensive analysis and it can facilitate the numerical analysis by determining the abnormality analysis criteria that can be exploited when defining how abnormalities should be considered in numerical analysis, such as when calculating the duration of double or oscillating peaks. To further analyze the mechanism behind different Ca^2+^ cycling abnormalities, combined electrophysiology and other functional methods together with Ca^2+^ imaging measurements are needed.

When Ca^2+^ signals are analyzed with *AnomalyExplorer*, the results are more comparable between different studies than they are with manual analysis because the user-defined anomaly detection parameters are specifically determined. Once the optimal user-defined parameters have been identified for specific measurement settings, there is no need for manipulating these parameters. With *AnomalyExplorer* exploiting the interactive visualization, it is easy to catch all of the abnormalities in a sample and obtain a more accurate and complete classification. In this study, we have shown that optimal analysis parameters can be found for data recorded with two different recording software programs and that the detection of normal and abnormal signals as well as the abnormality patterns with *AnomalyExplorer* is quite consistent with the manual analysis. Some variation in the manual and *AnomalyExplorer* analysis was observed in the categorization of abnormalities. When classifying the signals as normal or abnormal, inconsistency of the analysis could be seen in 2% and 9% of signals with Recording Software 1 and 2, respectively, which was quite reasonable. When categorizing the abnormalities into subgroups and calculating the number of different subgroup specific abnormalities, the abnormalities were detected with 0 to 5% difference between the manual and *AnomalyExplorer* analyses. The exception was in the amount plateau and low peak abnormalities in Recording Software 2 signals with 8 and 10% differences between manual and *AnomalyExplorer* analysis. When comparing manual and *AnomalyExplorer* analyses of different RyR2 mutated CPVT cell lines it was shown that detection of oscillation and middle peaks were equivalent with 0 to 5% differences in addition to double peak detection, which was quite similar between analyses with 0 to 5% differences in 4 out of 6 mutations. Detection of low peaks, irregular phases and plateau abnormalities differed the most between analyses. Overall, the inconsistency between manual and *AnomalyExplorer* analysis may be due to differences between human and software analyses because the accuracy of the manual analysis varies since it is performed only by visual recognition without clear abnormality detection rules. Higher percentages of low peak abnormalities were detected in manual analysis than in *AnomalyExplorer* analysis. This could be explained with background noise of the Ca^2+^ recordings, which is challenging to recognize visually in the manual analysis. Also irregularity of the beating rhythm as well as the prolonged rise and decay time (plateau abnormalities) of Ca^2+^ transients are challenging to detect visually in manual analysis since criteria for these abnormality types is difficult to define. With *AnomalyExplorer* the noise and abnormality detection rules are clearly defined and therefore the anomaly detection is more compatible between signals. Inconsistency between manual and *AnomalyExplorer* analysis can also be seen due to problems in the Ca^2+^ recording setup, such as with photobleaching of the Ca^2+^ indicator or its toxicity together with UV light to CMs, which can interfere with the loading of the cells and cause the transients and thus the whole signal to have extra noise. This can also affect the validity of *AnomalyExplorer* analysis and may be stated as one limitation of this study.

When comparing the analysis of different CPVT cell lines generated from patients carrying different RyR2 mutations the percentage of different abnormality subgroups varied between cell lines. Overall, double peaks and oscillations were most common and middle peaks and irregular phases least common abnormality types in all CPVT mutations. Other than that, no specific trend between abnormality subgroup percentages and different mutations could be seen and it could be concluded that there were mutation-specific differences in the Ca^2+^ transient abnormalities like we have previously shown with these cell lines [9].This finding showed the future usefulness of *AnomalyExplorer* for detecting cell line- and mutation-specific variability in the Ca^2+^ transient abnormalities. These types of differences reflect the mutation specific pathology of the disease and reveal that the location of the mutation can affect the disease phenotype. This result is in accordance with our previous study [9], where we showed that the Ca^2+^ abnormalities of these different CPVT cell lines are more common in some mutations than in others and that these Ca^2+^ cycling abnormalities can be rescued or reduced with a specific drug as in actual patients. In the future, *AnomalyExplorer* can speed up and improve the accuracy of the analysis of this type of drug study, where the effect of a specific type of drug on the amount Ca^2+^ cycling abnormalities is quantified. Further Ca^2+^ cycling studies combined with electrophysiological studies, are needed to assess how different Ca^2+^ cycling abnormalities reflect the mechanism of the disease or drug effect because these are not yet known. Presumably, at least some Ca^2+^ cycling abnormalities reflect the arrhythmogenic features such as DADs and EADs. Because electrophysiological studies are time-consuming and challenging and because only a restricted number of cells can be measured, these methods are not often combined and more optimization is needed.

*AnomalyExplorer* was designed for the data analysis of <50 Hz sampling frequency data with recording duration of tens of seconds, and it performs flawlessly under these requirements. *AnomalyExplorer* is able to analyze data with higher sampling frequencies, at least to 100 Hz, but it could be improved to suit for analysis of even higher frequency data in the future. With functional recordings it is problematic to set a certain recording duration limit that is sufficient to define the overall behavior of a cell and there is always uncertainty how well the recording reflects the overall behavior of the cell. In our study the recording duration was implemented for our specific use case with varying recording durations of 11 to 23 seconds. The analysis software does not have a limit for the recording length, but it can be estimated that responsiveness and user experience will suffer with several minute recordings. Short duration signals may not represent the long-term behavior of the cell correctly and for the future Ca^2+^ cycling analysis, the use of longer recordings with standard duration could be more beneficial, if it would be suitable for the study design. Ín these analyses, the duration of each abnormal Ca^2+^ transient could be compared to the duration of the whole signal to see the percentage of the abnormal Ca^2+^ transients in the recording. To improve the analysis of long-term behavior of the cells, long recordings could be also broken up into a series of segments and analyzed separately before averaging. Sampling frequency and recording duration of this study can be stated as a limitation of this study and the current prototype, as it was designed and implemented for a specific use case and for the specific digitization systems. Nevertheless, recording durations of tens of seconds and sampling frequencies of <50 Hz have been utilized successfully before, when studying Ca^2+^ cycling of iPSC derived disease specific CMs [2,5,9,23].

As the use of iPSC-derived CMs for disease modeling and drug screening continues to increase, there is a significant demand for faster and more consistent analysis methods. *AnomalyExplorer* is suitable for analyzing high number of Ca^2+^ signals, which can be a bottleneck for high-throughput Ca^2+^ imaging analysis and screenings in manual data analysis. This tool will facilitate and speed up the analysis of CM Ca^2+^ transients; furthermore it should be more accurate and user-independent. At present, we are not aware of any similar software that is suitable for single Ca^2+^ signal analysis that is recorded with any type of recording software. In the future, iPSC-derived CMs can be exploited for screening of potential new chemical compounds and this software and analysis method can be exploited for evaluating different drug responses and in Ca^2+^ cycling analysis to study basic disease pathology. This method will also be beneficial when analyzing Ca^2+^ cycling of CMs on a large scale for screening the adverse cardiac effects of new potential compounds.

## Conclusions

In this study, the *AnomalyExplorer* tool was developed to assist in analyzing and classifying Ca^2+^ signal abnormalities of disease specific human iPSC-derived CMs. Ca^2+^ cycling has a central role in CM contraction-relaxation, and it also is essential in the electrical signaling of CMs. As a result, detailed characterization of the abnormal Ca^2+^ transients present in different cardiac diseases would allow for a more detailed evaluation of the physiological function of the diseased CMs. Because Ca^2+^ transient abnormalities are difficult to specify and analyze by computational methods, in the *AnomalyExplorer* tool the abnormalities are recognized by the visual features alone, using interactive visualization with a similar approach as in traditional manual analysis. It is based on the multiple coordinated views paradigm and a direct manipulation UI. Using *AnomalyExplorer* the classifications are repeatable and user-independent. The software is also capable of screening large datasets faster than manual analysis. By utilizing fast kinetic fluorescence imaging of intracellular Ca^2+^ levels, together with *AnomalyExplorer*, the Ca^2+^ transient abnormalities of spontaneously contracting CMs can be detected and classified, which provides more information regarding the Ca^2+^ cycling phenotype in these cells.

## Supporting Information

S1 Fig Consistency of the manual analysis and *AnomalyExplorer* analysis. Totally 132 Ca^2+^ signals recorded with Recording Software 1 (A) and 212 Ca^2+^ signals recorded with Recording Software 2 (B) were analyzed.
(TIF)

S2 Fig Comparison of manual and *AnomalyExplorer* Ca^2+^ signal analysis of the data generated with Recording Software 1 and 2. A) Pie charts indicate the percentage of normal (white) and abnormal (black) Ca^2+^ signals in manual and *AnomalyExplorer* analyzed signals. B) Color-coded pie charts indicate the percentage of the different abnormalities in both manually and *AnomalyExplorer* analyzed signals. One Ca^2+^ signal can belong to several subgroups and consist of many abnormalities. In Recording Software 1 signals, totally 171 and 157 abnormalities were found in manual and *AnomalyExplorer* analysis, respectively. In Recording Software 2 signals, totally 301 and 284 abnormalities were found in manual and *AnomalyExplorer* analysis, respectively.
(TIF)

S1 Supporting Information Availability of software including link to the software licensed under MIT, documentation for running and installing the software, and a test dataset for running the software. (DOCX)
