# Supplementary figures and images for: Novel Analysis Software for Detecting and Classifying Ca2+ Transient Abnormalities in Stem Cell-Derived Cardiomyocytes
Source: PLoS One. 2015 Aug 26;10(8):e0135806. doi: 10.1371/journal.pone.0135806 (PMC4550257; doi:10.1371/journal.pone.0135806)

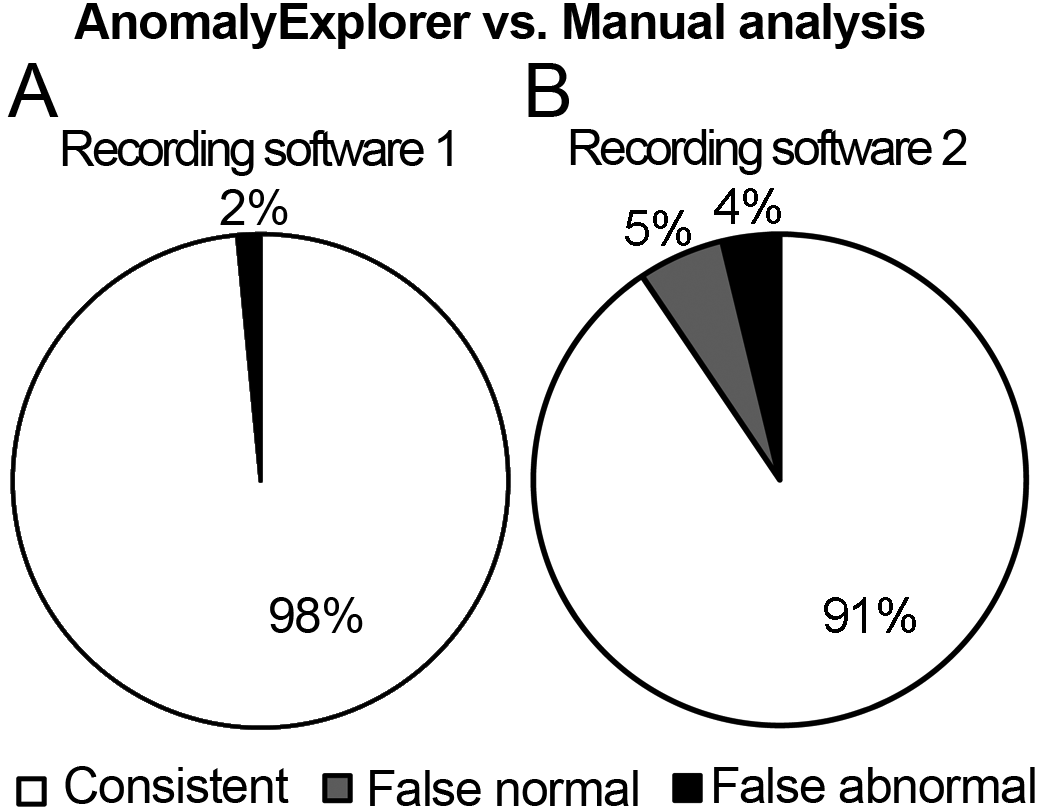

Supplement: S1 Fig — Totally 132 Ca2+ signals recorded with Recording Software 1 (A) and 212 Ca2+ signals recorded with Recording Software 2 (B) were analyzed. (TIF) [file pone.0135806.s001.tif]

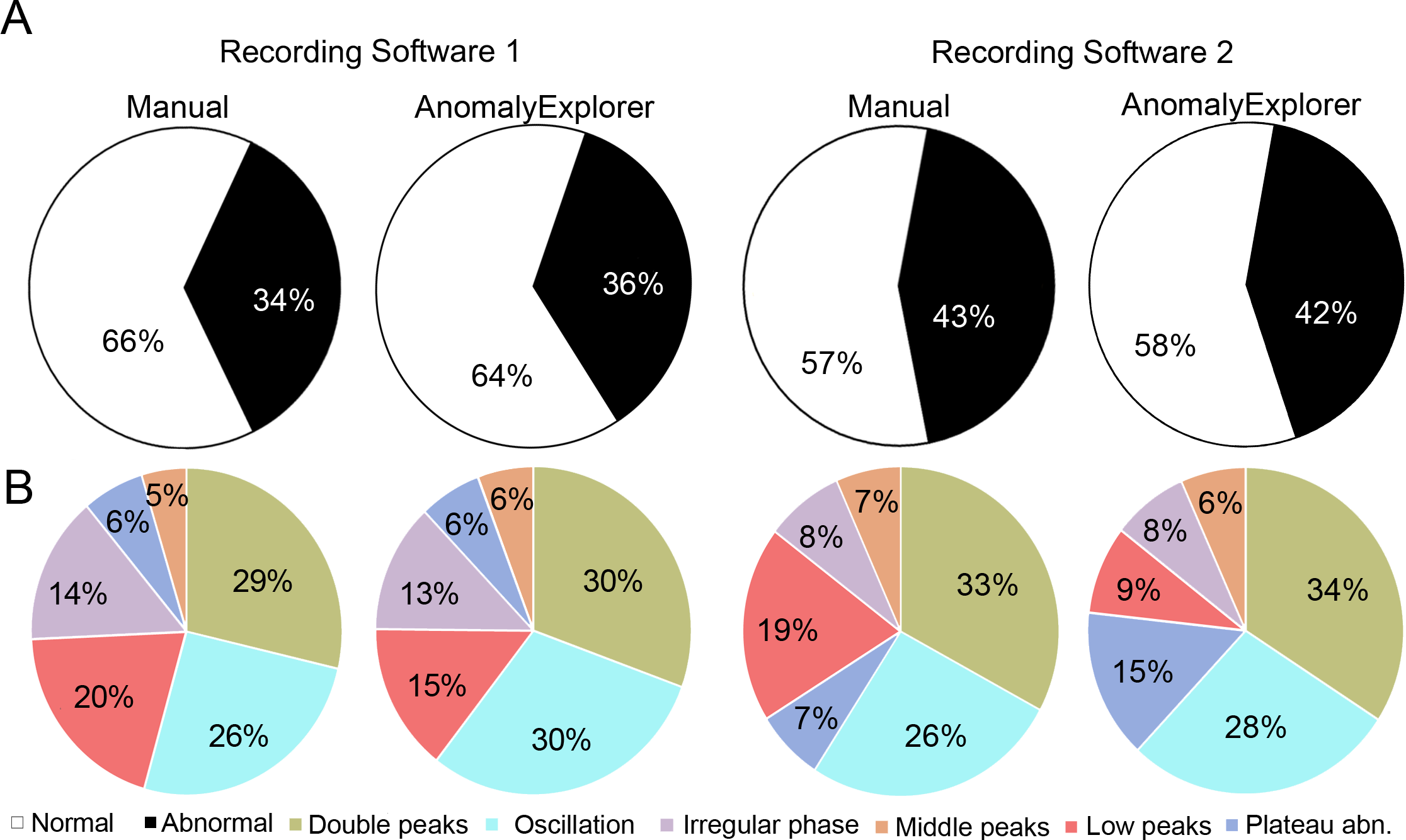

Supplement: S2 Fig — A) Pie charts indicate the percentage of normal (white) and abnormal (black) Ca2+ signals in manual and AnomalyExplorer analyzed signals. B) Color-coded pie charts indicate the percentage of the different abnormalities in both manually and AnomalyExplorer analyzed signals. One Ca2+ signal can belong to several subgroups and consist of many abnormalities. In Recording Software 1 signals, totally 171 and 157 abnormalities were found in manual and AnomalyExplorer analysis, respectively. In Recording Software 2 signals, totally 301 and 284 abnormalities were found in manual and AnomalyExplorer analysis, respectively. (TIF) [file pone.0135806.s002.TIF]
