# Supplementary material for: Novel Analysis Software for Detecting and Classifying Ca2+ Transient Abnormalities in Stem Cell-Derived Cardiomyocytes
Source: PLoS One. 2015 Aug 26;10(8):e0135806. doi: 10.1371/journal.pone.0135806 (PMC4550257; doi:10.1371/journal.pone.0135806)
Supplement: S1 Supporting Information — (DOCX) [file pone.0135806.s003.docx]

**S1 Supporting Information Penttinen et al.**

**Open Software Archive PONE-D-15-10710**

**AnomalyExplorer software**

The AnomalyExplorer (AE) is a HTML5-based analysis tool for calcium cycling. It is based on the earlier Java implementation (Siirtola et al. 2014).

AE is a one-page web application implemented mainly in JavaScript. It should run on any modern web browser, but it has been specifically tested with Google Chrome and Safari. Software conforms the Open Source Definition.

**The Latest Version**

AE can be found on the AnomalyExplorer project page under <https://github.com/siirtola/AnomalyExplorer>

**Documentation**

Currently, the only documentation is the accepted PLOS-One article. The distribution contains test dataset files demonstrating the anomaly types that can be detected with AE. The most up-to-date documentation can be found at https://github.com/siirtola/AnomalyExplorer

**Installation**

AE does not require any installation beyond copying the test dataset files and the folder structure into a new location. AE does not require a web server as it can be run from a local copy.

**Running of the software**

Files include test dataset, which can be uploaded to AE from “Choose files” function. Suitable control parameter settings for this test dataset are set as default but can be as well controlled by user. Immediately after the files to be analyzed are uploaded to the software, results with the default parameter settings are shown.

**Licensing**

AE is distributed under MIT license.

**Contacts**

- For questions related to biomedical aspects of AE,

please contact Kirsi Penttinen, [kirsi.penttinen@uta.fi](mailto:kirsi.penttinen@uta.fi)

- For questions related to technological aspects of AE,

please contact Harri Siirtola, [harri.siirtola@uta.fi](mailto:harri.siirtola@uta.fi)

**References**

Siirtola, H., Àvalos-Salguero, J., Penttinen, K., Aalto-Setälä̈, K., and Juhola, M. (2014). Interactive biosignal analysis and classification. In Information Visualisation (iV2014), 18th International Conference, pages 327–332. (PDF available upon request.)
